# Contagious Deposition of Seeds in Spider Monkeys' Sleeping Trees Limits Effective Seed Dispersal in Fragmented Landscapes

**DOI:** 10.1371/journal.pone.0089346

**Published:** 2014-02-27

**Authors:** Arturo González-Zamora, Víctor Arroyo-Rodríguez, Federico Escobar, Matthias Rös, Ken Oyama, Guillermo Ibarra-Manríquez, Kathryn E. Stoner, Colin A. Chapman

**Affiliations:** 1 División de Posgrado, Instituto de Ecología A.C., Xalapa, Veracruz, Mexico; 2 Centro de Investigaciones en Ecosistemas, Universidad Nacional Autónoma de México, Morelia, Michoacán, Mexico; 3 Red de Ecoetología, Instituto de Ecología A.C., Xalapa, Veracruz, Mexico; 4 Escuela Nacional de Estudios Superiores, Universidad Nacional Autónoma de México, Morelia, Michoacán, Mexico; 5 Department of Fish, Wildlife, and Conservation Ecology, New Mexico State University, Las Cruces, New Mexico, United States of America; 6 Department of Anthropology and McGill School of Environment, McGill University, Montreal, Quebec, Canada and Wildlife Conservation Society, Bronx, New York, United States of America; Key Laboratory of Tropical Forest Ecology, Xishuangbanna Tropical Botanical Garden, Chinese Academy of Sciences, China

## Abstract

The repeated use of sleeping sites by frugivorous vertebrates promotes the deposition and aggregation of copious amounts of seeds in these sites. This spatially contagious pattern of seed deposition has key implications for seed dispersal, particularly because such patterns can persist through recruitment. Assessing the seed rain patterns in sleeping sites thus represents a fundamental step in understanding the spatial structure and regeneration of plant assemblages. We evaluated the seed rain produced by spider monkeys (*Ateles geoffroyi*) in latrines located beneath 60 sleeping trees in two continuous forest sites (CFS) and three forest fragments (FF) in the Lacandona rainforest, Mexico. We tested for differences among latrines, among sites, and between forest conditions in the abundance, diversity (α-, β- and, γ-components) and evenness of seed assemblages. We recorded 45,919 seeds ≥5 mm (in length) from 68 species. The abundance of seeds was 1.7 times higher in FF than in CFS, particularly because of the dominance of a few plant species. As a consequence, community evenness tended to be lower within FF. β-diversity of common and dominant species was two times greater among FF than between CFS. Although mean α-diversity per latrine did not differ among sites, the greater β-diversity among latrines in CFS increased γ-diversity in these sites, particularly when considering common and dominant species. Our results support the hypothesis that fruit scarcity in FF can ‘force’ spider monkeys to deplete the available fruit patches more intensively than in CFS. This feeding strategy can limit the effectiveness of spider monkeys as seed dispersers in FF, because (i) it can limit the number of seed dispersers visiting such fruit patches; (ii) it increases seed dispersal limitation; and (iii) it can contribute to the floristic homogenization (i.e., reduced β-diversity among latrines) in fragmented landscapes.

## Introduction

Seed dispersal processes link the reproductive cycle of adult plants with the establishment of their offspring [1]. Assessing the patterns of seed rain thus represents a fundamental step to understand the spatial structure and regeneration of plant populations, and is critical in understanding patterns of species richness [2]. In the tropics, more than 60% and up to 94% of woody plant species have their seeds dispersed through endozoochory [3] and primates are among the most prominent taxa of seed-dispersing frugivores [4]. Although many primates deposit copious amounts of seeds in latrines beneath sleeping trees, little is known about the ecological implications of this spatially contagious pattern of seed deposition [4].

Schupp et al. [5] argue that contagious seed dispersal can reduce the quality of dispersal because it creates dissemination limitation for other potential plant recruitment sites, and consequently recruitment limitation. Furthermore, based on the Janzen–Connell hypothesis [6], [7], seed/seedling mortality could be higher in latrines, since the aggregation of seeds can attract predators and/or pathogens that act in a density-dependent fashion. Nevertheless, growing empirical evidence demonstrates that primate latrines are enriched in nutrients compared to surrounding areas [8], [9] and such soil enrichment can positively affect the establishment, growth, and survival of seedlings arising from primate-dispersed seeds [4], [10], [11]. Thus, consistent with the ‘directed dispersal hypothesis’ [12], primate latrines can represent non-random habitats, where survival of seeds and seedlings could be relatively high. Therefore, assessing the seed rain patterns in primate latrines is a fundamental task for understanding the potential impacts that latrines have on the spatial distribution of plant populations, as well as on emerging properties, such as community structure and diversity [4], [13].

In terms of seed dispersal quantity (sensu Schupp [14]), spider monkeys (*Ateles* spp.) likely represent one of the most effective seed dispersers in Neotropical rainforests, as there is no other mammal dispersing higher quantities of seeds per kilogram of biomass [15], [16]. Spider monkeys are specialized frugivores that incorporate a diverse array of fruit species in their diets (e.g., 152 plant species by *A. belzebuth* [17]; 165 species by *A. geoffroyi* [18]). The seeds of most of these plant species are swallowed [17], [19], and are then defecated following a mixed seed deposition pattern. A fraction of these seeds are deposited during the day in individual scats distributed across the forest and the remaining seeds are deposited at night or early morning in one or more latrines beneath sleeping sites [19]–[21]. Although a few studies have described the use, availability, and spatial distribution of spider monkey latrines [20], [22], [23], to our knowledge no study to date has assessed the abundance, species diversity, and/or composition of seeds that fall within these sites. Furthermore, spider monkeys are increasingly forced to inhabit fragmented landscapes [24], [25], but it is virtually unknown how the seed rain patterns produced by these primates will alter the future tree composition of these fragments.

**Figure 1:**
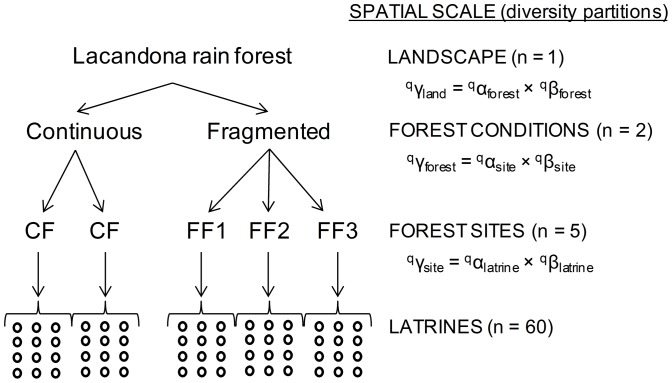
Hierarchically nested sampling design. The figure shows the spatial scales used to assess differences in species diversity of seeds defecated by spider monkeys (*Ateles geoffroyi*) in latrines located in continuous and fragmented forest in the Lacandona region, Mexico. Seed species diversity was partitioned into α- and β-components considering three spatial scales, from larger to smaller: (i) the diversity of the landscape (γ_land_) was partitioned into mean alpha (α_forest_) and beta (β_forest_) diversities in the two forest conditions; (ii) the diversity within each forest condition (γ_forest_) was partitioned into mean alpha (α_site_) and beta (β_site_) diversities in the sites; and (iii) the diversity within each site (γ_site_) was partitioned into mean alpha (α_latrine_) and beta (β_latrine_) diversities in the 12 sampling latrines.

Based on a hierarchically nested sampling design (Figure 1), we assessed the seed rain produced by spider monkeys (*Ateles geoffroyi*) in 60 latrines located in two continuous forest sites and three forest fragments in the Lacandona rainforest, Mexico. Using a multiplicative diversity partitioning approach, we assessed variations among latrines, among sites and between forest conditions in the abundance, diversity (α-, β- and γ-components), and evenness of seed assemblages (Figure 1). The species diversity was evaluated using true diversity measures (i.e., numbers equivalents); an analytical approach that has been recently recognized as the most appropriate for diversity evaluations [26], [27]. We considered true diversities ^0^D (species richness), ^1^D (exponential of Shannon's entropy) and ^2^D (inverse Simpson concentration). ^0^D is not sensitive to species abundances and so gives disproportionate weight to rare species [26]. ^1^D weights each species according to its abundance in the community, and hence, it can be interpreted as the number of ‘common’ species in the community [28]. Finally, ^2^D favors abundant species, and can be actually interpreted as the number of ‘very abundant’ or ‘dominant’ species in the community [28]. Thus, we identified the abundance level, from rare to common to dominant species, at which we observed higher variations in seed species diversity across different spatial scales.

Because fruit availability can vary widely among sites, and spider monkeys can adapt their diet to food availability within each site [16], [18], [30], we hypothesized that patterns of abundance, diversity, and evenness of seed assemblages will be highly variable among latrines. In particular, fruit availability is typically lower in fragments than in continuous forest because of the combination of both smaller home range sizes [30] and a lower density of big (dbh>60 cm) food trees (i.e., larger fruit patches [31]) in fragments [32], [30], [33]. Thus, the abundance and species diversity of seeds within latrines is expected to be lower in fragments where spider monkeys usually spend more time consuming leaves [18], [30], and the number of feces without seeds is usually higher than in continuous forests [19]. However, we also predict that fruit scarcity in fragments will ‘force’ spider monkeys to spend more time consuming the available fruit patches; i.e., they will deplete the available patches more intensively than in continuous forest sites [34], [35]. As consequence, the seed rain in fragments will be dominated by a few plant species, reducing the number of common (^1^D) and dominant species (^2^D), as well as the seed community evenness in forest fragments.

## Materials and Methods

### Ethics Statement

This study adhered to the laws of the Mexican Government (SEMARNAT, Secretaría de Medio Ambiente y Recursos Naturales) to work with wild animals and plants in Lacandona (permit no. SGPA/DGVS/09606). Since our work is not invasive, only observational, we meet all ethical and legal requirements established by the American Society of Primatologists (ASP), Animal Care and Use Committee, and Ethical Committee of the Zoological Society of London for work on primates. Although our institution, Universidad Nacional Autónoma de Mexico (UNAM), does not yet have an Institutional Review Board (IRB) or a similar governing body of ethics, this project was approved by the Consejo Nacional de Ciencia y Tecnología (project CB-2006-56799). We thank the owners of the forest patches for giving us the permission to perform the research in the study sites.

### Study Site

The Lacandona rainforest constitutes the southwestern sector of the Mayan forest in Mexico, and it is one of the most important rainforest remnants in Mesoamerica. The area is located in the northeastern portion of the state of Chiapas, and is delimited by the Guatemalan border on the south and east, and by the Chiapas highlands on the north and west. Average monthly temperatures range from 24°C to 26°C, and mean annual rainfall is 2,500–3,500 mm, with roughly 80% of the rains falling between June and November. The area was originally covered by over 1.4 million ha of rainforest, but human settlement and deforestation between 1960 and 1990 resulted in the loss of 70% of the original forest cover.

We worked in two adjacent areas separated by the Lacantún River (>150 m wide): the Marqués de Comillas region (MCR, eastern side of the river) encompassing ca. 176,200 ha of fragmented forest, human settlements, and agricultural lands. Approximately 50% of the land surface of MCR is now used for cattle ranching and agriculture, but several fragments (0.5–1,500 ha) remain. The second area was the Montes Azules Biosphere Reserve (MABR, western side) comprising ca. 331,000 ha of undisturbed old-growth forest.

### Experimental Design and Indicators of Food Availability

Based on a recent study on the density and spatial distribution of sleeping sites and latrines of spider monkeys (*Ateles geoffroyi*) in four continuous forest sites within MABR and four forest fragments in MCR [23], we selected sites with more than 12 latrines (i.e., three fragments and two continuous forest sites) to control for sampling effort (i.e., we sampled 12 latrines per site, see details below). The continuous forest sites were separated by at least 5 km from each other (CF1: 16°06′25.01” N – 91°59′16.61” O; CF2: 16°06′50.25” N – 90°56′24.46” O). The fragments were isolated ≥24 yrs ago, are immersed in an anthropogenic matrix, and their distances to continuous forest ranged from 200 to 1,200 m (FF1: 16°15′10.83” N – 90°49′53.82” O; FF2: 16°16′54.15” N – 90°50′19.91” O; FF3: 16°19′54.85” N – 90°51′10.71” O). The average isolation distance among fragments is 4,200 m (a detailed map of the sites is located in [23]).

Tree species diversity was similar in continuous and fragmented forests, both when considering the whole tree community (i.e., trees with diameter at breast height, dbh ≥10 cm) and when considering the top spider monkey food tree species (i.e., those contributing to >80% of total feeding time in a recent review of spider monkey diet in Mesoamerica [18]; Figure S1). However, the density (stems/1,000 m^2^) and basal area of top food species were significantly higher in continuous than fragmented forest sites (Appendix S1 and Table S1). Thus, as previously reported for this [30] and other Mexican rainforests [32], food availability can be limited in fragments, as the lack of large food trees can limit the availability of fruits [31].

### Seed Collection

Within each site we randomly selected 12 latrines (60 in total). We measured the seed rain within each latrine for 13 months (February 1, 2011 to February 28, 2012) by placing one seed trap in the center of each latrine. Each seed trap consisted of a circular 1.5-m diameter PVC frame supporting a 0.5-m depth, open-topped, 0.5-mm nylon mesh bag suspended 1 m above the ground on three thin steel posts to prevent predation by terrestrial vertebrates. The continuous falling of leaves and dung also contributed to hide seeds, thus further reducing the probability of seeds being removed by animals. In fact, we did not detect signs of seed predation (e.g., open husks, seeds with teeth marks) within the traps. Traps were emptied once a month and the seeds located within the spider monkeys' feces were collected, washed, counted, and identified to species level based on (i) our experience with the local flora [19], [30]; (ii) the knowledge of local parataxonomists; and (iii) information from seed catalogs [36]. Only seeds ≥5 mm in length were recorded. Although seed traps also captured some fruits and seeds dispersed by wind or gravity, we only considered seeds immersed within monkeys' feces. These were identified in the field based on their typically “stained” appearance and characteristic adhesion of fecal matter.

### Data Analyses

We first evaluated sample completeness within each latrine in the following manner [29]:
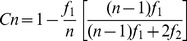
where f_1_ and f_2_ are the number of species represented by one (singletons) and two (doubletons) individuals in the sample, respectively, and n is the total number of individuals in the sample. Sample coverage did not differ between sites (Kruskal-Wallis test, *H* = 6.7, *P* = 0.14), averaging (± SD) 99%±1% (range = 93–100%) per latrine, indicating that the seed inventory was accurate with our sampling effort, and that our results are not biased by differences in sample completeness among sites.

Based on our hierarchically nested sampling design (i.e., 60 latrines in 5 sites within two forest conditions in one landscape; Figure 1), we analyzed patterns of seed species diversity across multiple spatial scales using Hill numbers (^q^D). These metrics represent true diversities because they obey the replication principle [27]. They are in units of ‘species’, which facilitates comparison between samples. It is thus possible to plot them all on a single graph to compare diversity profiles as a continuous function of the parameter q. This ‘diversity profile’ characterizes the species–abundance distribution of a community and provides complete information about its diversity [27]. For S species and 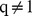, Hill numbers of order q are defined as:
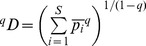
where p_i_ indicates the relative abundance of the ith species, and q is an exponent that determines the sensitivity of the measure to the relative abundances. Because the Hill number is undefined for q = 1, the diversity of order 1 can be estimated as:
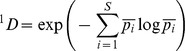
We considered three orders for q (0, 1, and 2) in its unweighted form [27]. ^0^D is the species richness, ^1^D is equivalent to the exponential of Shannon's entropy, and ^2^D is equivalent to the inverse Simpson concentration [26]. When considering several communities, alpha and gamma components of diversity can be analyzed following Jost [27]:
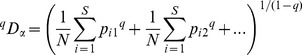
where p_i_ denotes the relative abundance of the ith species in each of the N communities. Again, for the particular case of q = 1, α-diversity can be estimated as:
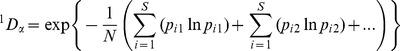



Then, using a multiplicative partitioning of Hill numbers, beta (between group) component of diversity can be calculated as: ^q^D_β_ = ^q^Dγ/^q^D_α_. This beta can be interpreted as the ‘effective number of completely distinct communities’ [27], which ranges from one (when all communities are identical) to N (when all communities are completely distinct).

To evaluate changes in different components of diversity (γ, α, and β) at multiple spatial scales, we partitioned species diversity into within- (α) and between- (β) components considering three spatial scales (Figure 1): (i) the diversity of the landscape (γ_land_) was partitioned into mean alpha and beta diversities in the two forest conditions (^q^γ_land_ = ^q^α_forest_×^q^β_forest_); (ii) the diversity within each forest condition (γ_forest_) was partitioned into mean alpha and beta diversities in the sites (^q^γ_forest_ = ^q^α_site_×^q^β_site_); and (iii) the diversity within each site (γ_site_) was partitioned into mean alpha and beta diversities in the 12 sampling latrines (^q^γ_site_ = ^q^α_latrine_×^q^β_latrine_). To assess if the magnitude in β-diversity differed between forest conditions, we compared the relative compositional dissimilarity between communities using the transformation of beta (^q^D_β_) proposed by Jost [26] for communities with different numbers of samples (i.e., continuous forest: n = 2; fragments: n = 3): ^q^DS = 1 – [(1/^q^D_β_-1/N)/(1-1/N)], where N is the number of samples. ^q^DS = 1, when all the samples are completely distinct, and ^q^DS = 0, when all are identical.

We also calculated changes in species dominance across spatial scales using the evenness factor proposed by Jost [28]: EF_0,2_ = ^2^D/^0^D. This measure was used because it: (i) is calculated from true diversity measures; (ii) is independent of the number of species in the sample; and (iii) is very easy to interpret. This index ranges between 1 (when all species are equally common) and nearly 1/S (when the community is totally dominated by one species) [28]. Roughly speaking, EF can be interpreted as the proportion of dominant species in the community [28].

To assess if seed species diversity and abundance differed among forest conditions, we used generalized linear models. As suggested for count dependent variables (i.e., ^0^D and abundance of seeds), we used a Poisson error and a log link function. For EF, ^1^D and ^2^D we used normal error and an identity link function [37]. To assess if latrines can be considered independent samples, we applied a Mantel test using the XLSTAT program (version 2012.6.08) to correlate the compositional similarity among latrines (Bray-Curtis index) with the inter-latrine isolation distances (ln-transformed). The Mantel-test detected a significant spatial autocorrelation of data sets (*R* = −0.423, *P* = 0.0001), thus, we cannot consider the latrines as replicates for testing differences among sites. Therefore, differences in species diversity and abundance among sites were tested using general linear mixed models (GLMM) with JMP 8.0, where the fixed effect was "sites". To control for the unavoidable pseudoreplication effect of our design, we nested latrines within each site as a random effect in the models. Residual maximum likelihood method (REML) was used to separate variances of fixed from random effects in the models [38].

## Results

We recorded 45,919 seeds belonging to 32 families, 49 genera, and 68 plant species (including 8 morphospecies) during the 13-mo period. The species with greater number of seeds were the palm *Sabal mexicana*, Arecaceae (13.1% of all records), the trees *Dialium guianense*, Fabaceae (12.6%), *Castilla elastica*, Moraceae (9.2%), *Spondias radlkoferi*, Anacardiaceae (6.3%), and *Trophis mexicana*, Moraceae (5.2%), and the lianas *Rourea glabra*, Connaraceae (5.1%), and *Paullinia costata*, Sapindaceae (4.7%). At the family level, most seeds were from Arecaceae (22.7%), Moraceae (15.4%), Fabaceae (15.4%), Anacardiaceae (8.8%), Sapindaceae (5.5%), and Connaraceae (5.1%), together representing 72.9% of all seeds recorded (Table S2).

### Abundance of Seeds and Species Diversity across Scales

**Figure 2:**
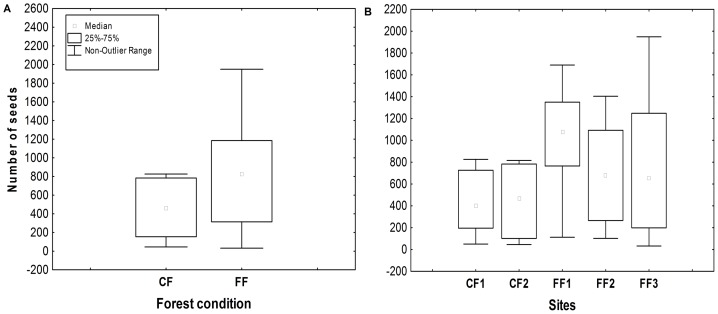
Abundance of seeds deposited by spider monkeys in latrines located in continuous and fragmented forests in the Lacandona region, Mexico. We show differences between forest conditions considering medians per site (a), and among sites based on medians per latrine (b). FF  =  forest fragments ordered from the largest to the smallest; CF  =  continuous forest sites.

The abundance of seeds was highly variable among sites, ranging from 6,234 seeds in CF1 to 15,414 seeds in FF1. Seeds were 1.7 times more abundant in fragments (mean ± SE, 11,045±3,853 seeds) than in continuous forest sites (6,393±224 seeds) (*χ^2^* = 3.07, df = 1, *P* = 0.08; Figure 2a). The mean number of seeds per latrine was 765 (ranging from 32 to 4,621 seeds), and tended to differ among sites (*F*_4,55_ = 2.34, *P* = 0.06), being between 1.6 and 2.5 times higher in FF1 than in the rest of the sites (Figure 2b).

**Figure 3:**
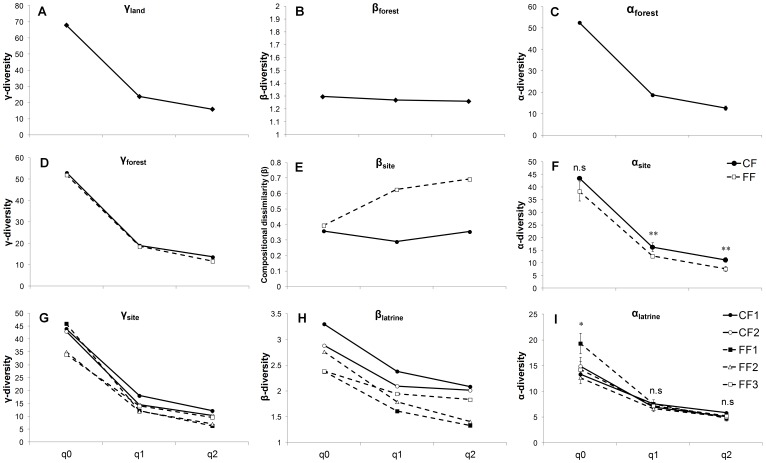
Seed species diversity in spider monkeys' latrines located in continuous and fragmented forests in the Lacandona region, Mexico. From left to right, the panels show γ-, β-, and α-components of diversity at three spatial scales. The diversity of the landscape (γ_land_; panel a) was partitioned into mean β- (b) and α- (c) diversities within the two forest conditions. The diversity within each forest condition (γ_forest_; panel d) was partitioned into mean β- (e) and α- (f) diversities in the sites. Finally, the diversity within each site (γ_site_; panel g) was partitioned into mean β- (h) and α- (i) diversities in latrines. Mean (± SE) α-diversities per forest condition, per site and per latrine is indicated in panels c, f and i, respectively (in panels f and i, significant differences are indicated with asterisks; * *P*<0.05; ** *P*<0.01; n.s. *P*>0.05). In all cases, we evaluated true diversities of order 0 (species richness), 1 (exponential of Shannon's entropy), and 2 (inverse Simpson concentration); however, in panel e we compared the relative compositional dissimilarity between forest conditions using the transformation of beta proposed by Jost (2007) for communities with different numbers of samples (CF: *n* = 2; FF: *n* = 3) (see Materials and Methods).

At the landscape scale, total species diversity (γ_land_) was, on average, 1.28 times higher than mean species diversity per forest condition (α_forest_) for any order of q, as species turnover between forest conditions (β_forest_) was almost the same (1.26 to 1.30) for all q orders (Figures 3a–c). When analyzing each forest condition separately, mean species diversity per site (α_site_) was similar in continuous and fragmented forests for ^0^D (*χ^2^* = 1.05, df = 1, *P* = 0.30), but was significantly higher in continuous forest than in fragments in terms of *^1^D* (*χ^2^* = 8.58, df = 1, *P* = 0.003) and *^2^D* (*χ^2^* = 10.0, df = 1, *P* = 0.001; Figure 3f). Nevertheless, since species turnover (β_site_) was two times greater among fragments than between continuous forest sites when considering ^1^D and ^2^D (Figure 3e), the accumulated number of species (γ_forest_) was almost the same in continuous and fragmented forests (Figure 3d). Finally, at the site scale, mean species diversity per latrine (α_latrine_) differed among sites for ^0^D (*F*_4,55_ = 2.73, *P* = 0.04), being significantly higher in the largest fragment (FF1) than in the rest of the sites; however, mean ^1^D and ^2^D per latrine did not differ among sites (*P*>0.68 in all cases) (Figure 3i). Species turnover among latrines (β_latrine_) was notably higher in continuous forest sites than in fragments for any order of q (Figure 3h), and as consequence, in most cases the continuous forest sites accumulated a greater number of species (γ_site_) than fragments (Figure 3g).

### Community Evenness across Spatial Scales

**Figure 4:**
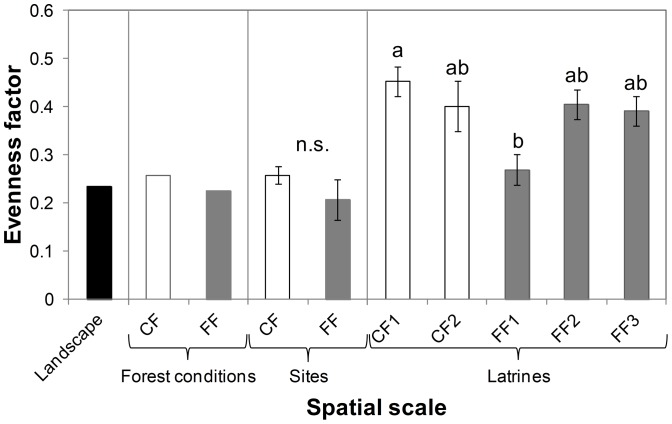
Species evenness in seeds deposited by spider monkeys in latrines located in continuous and fragmented forests in the Lacandona region, Mexico. Differences across spatial scales are indicated; from the landscape scale (i.e., including both forest conditions) to the latrine scale. Means (± SE) per site and per latrine are indicated for the site and latrine spatial scales. Significant differences among sites are indicated with different letters (*P* = 0.01). The evenness factor did not differ between forest conditions (n.s., *P*>0.05).

**Figure 5:**
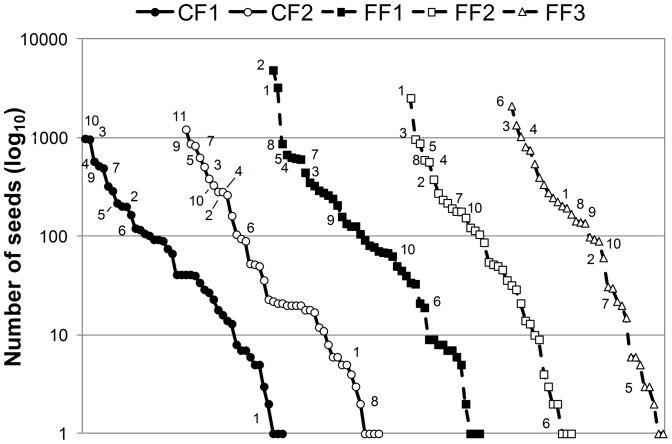
Relative abundance of seeds deposited by spider monkeys in latrines located in each study site. The identity of dominant species within each site is indicated: 1. *Sabal mexicana*; 2. *Dialium guianense*; 3. *Castilla elastica*; 4. *Spondias radlkoferi*; 5. *Trophis mexicana*; 6. *Rourea glabra*; 7. *Paullinia costata*; 8. *Bactris mexicana*; 9. *Trichostigma octandrum*; 10. *Ampelocera hottlei*; 11. *Mendoncia retusa*.

The evenness factor at the landscape scale (i.e., based on γ_land_) was 0.24 (Figure 4). At the forest condition scale (i.e., based on γ_forest_), the evenness factor was slightly higher in continuous (EF_0,2_ = 0.26) than in fragmented (EF_0,2_ = 0.23) forests. Based on means (± SE) per site (γ_site_), we also found a slightly higher evenness factor in continuous forest (0.26±0.02) than in fragments (0.21±0.04), but this difference was not significant (*χ^2^* = 1.37, df = 1, *P* = 0.24; Figure 4). This pattern was evident when analyzing the rank-abundance curves, which showed that in fragments the seed rain was dominated by 9 species, whereas in the continuous forest it was dominated by 5 species (Figure 5). In continuous forest sites, *C. elastica* and *Ampelocera hottlei*, and the lianas *Trichostigma octandrum*, *Paullinia costata*, and *Mendoncia retusa* represented 53.4% of all seeds recorded. However, in fragments, the palm *S. mexicana*, the trees *D. guianense*, *C. elastica*, and *S. radlkoferi*, and the liana *R. glabra* represented 55.1% of all seeds recorded (Table S2). The number of rare species followed the opposite pattern, being higher in continuous (*n* = 11 species) than fragmented forests (*n* = 8 species; Figure 5).

At the latrine scale, we found significant differences in evenness among sites (*F*_4,55_ = 3.50, *P* = 0.01; Figure 4), with the fragment FF1 showing lower evenness than the continuous forest CF1 (Figures 4 and 5). In CF1 the trees *A. hottlei* and *C. elastica* represented 31.3% of all seeds recorded whereas in CF2 the lianas *M. retusa*, *T. octandrum* and *P. costata* represented 44.6% of recorded seeds. Regarding the fragments, in FF1, the palm *S. mexicana* and the tree *D. guianense* represented 52.6% of all seeds recorded. In FF2, the palm *S. mexicana* and the trees *C. elastica* and *T. mexicana* represented 53.6% of all seeds recorded. Finally, in FF3, the liana *R. glabra* and the trees *C. elastica* and *Nectandra ambigens* represented 46.9% of all recorded seeds (Figure 5).

## Discussion

### Potential Causes of Seed Dispersal Patterns across Scales

Our results support the hypothesis that fruit scarcity in fragments (see Methods and Appendix S1) can result in spider monkeys depleting the available fruit patches more intensively than in continuous forest sites [34]. As predicted, the abundance of seeds was 1.7 times higher in fragments than in continuous forest sites. This was principally associated with the dominance of a few plant species, which tended to reduce seed community evenness in fragments. In particular, *Sabal mexicana* and *Dialium guianense* were by far the most abundant species in fragments providing seeds, which together represented 34% of all seeds at these sites (Table S2). The fruits from these species also are among the most commonly consumed by spider monkeys in these fragments [30], most likely because they are particularly abundant in fragments in this region (VAR, unpubl. data), and because they produce large amounts of fruits over long periods (i.e., March to August [39]). In fact, these two species were the most abundant in FF1 and FF2 (Table S1); the fragments in which these species were particularly common in the seed rain (Figure 5; Table S2). Therefore, in agreement with previous studies that have documented that spider monkeys can adapt their diet to resource availability [16], [30], our results suggest that to cope with a lower availability of food resources in fragments, this primate spends more time feeding on fruits from a few largely available and productive plant species.

This hypothesis was also supported by the fact that, considering common (^1^D) and dominant (^2^D) seed species, the compositional dissimilarity (β-diversity) among fragments was two times higher than between continuous forest sites (Figure 3e). Spider monkeys in continuous forest areas can have access to a greater amount of top food trees, and hence, they can feed from preferred foods. In this sense, 50% of the top species (i.e., those representing 75% of the total seed rain within each site) were the same in both continuous forest sites. However, in fragments, where primates need to adapt their diet to the available foods [16], [30], [33], the percentage of top species that were shared between pairs of fragments averaged 35%. This higher species turnover among fragments may be largely due to the fact that plant species composition strongly differs among fragments [40], not only because of greater inter-fragment isolation distances that can limit the interchange of plant species in fragmented forests [41], but also because of the differences among fragments in disturbance regimes (e.g., edge effects, logging), that are known to influence plant community composition [40], [42], [43]. Thus, the species turnover in the seed rain is most likely associated with the species turnover in the available food plant communities, particularly in terms of common and dominant fruit species.

At smaller spatial scales, it was particularly interesting that β-diversity among latrines was notably lower in fragments than in continuous forest sites. This seed community homogenization can be related to the fact that inter-latrine distances are almost double in continuous forest than in fragments [23]. This distribution of sleeping sites limits the availability of food resources they can obtain in fragments, as these primates are multiple-central place foragers (sensu [44]); i.e., they feed on different trees located in the vicinity of sleeping sites, and return to the same sleeping sites after their foraging excursions. Thus, the probability of sharing the same foraging areas, and food trees, by different subgroups of spider monkeys is probably higher in fragments than in continuous forests. This can explain the compositional homogenization of the seed rain among latrines and the sharp increase in the abundance of a few plant species in some fragments.

### Implications for Seed Dispersal and Forest Regeneration

Although these feeding strategies may allow primates to maintain their fruit diet in forest fragments, it may alter their effectiveness as seed dispersers in fragments. For example, in terms of dispersal quality (sensu [14]), spider monkeys appeared to deplete the fruit patches more intensively in fragments than in continuous forest sites. This can reduce the probability that such plant species are dispersed by other high-quality dispersers (e.g., howler monkeys, large birds, frugivorous bats). From the plant point of view, the higher the number of seed dispersers, the greater the probability of creating complex composite seed shadows and establishing seedlings in a larger number of suitable sites [13], [45]. Additionally, seed dispersal limitation can also result directly from the deposition of a large number of seeds in latrines [5]. For example, spider monkeys deposited 4,868 seeds of *D. guianense* in fragment FF1, 2,539 seeds of *S. mexicana* in FF2 and 2,115 seeds of *Rourea glabra* in FF3; whereas dominant species in continuous forest sites showed a notably lower number of seeds (988 seeds of *Ampelocera hottlei* in CF1, and 1,220 seeds of *Mendocia retusa* in CF2). Although the accumulation of seeds in latrines could saturate seed predators and therefore allow some seeds to escape predation and recruit near latrines [11], this seed dispersal pattern clearly limits the dissemination to other potential plant recruitment sites [5]. Furthermore, because the distance among primates' sleeping sites can be a good indicator of seed dispersal distances [16], dispersal limitation is expected to be higher in fragments, in which sleeping sites are closer together [23]. In this sense, the combination of reduced inter-latrine distances in fragments and a higher abundance of seeds in latrines from these forest remnants can increase the incidence of density-dependent mortality factors (e.g., seed predators, pathogens) [6], [7], limiting the establishment and survival of seedlings in latrines from fragments.

Finally, our results indicate that primates in fragments can contribute to plant community homogenization, limiting the total number of species (γ-diversity) that they can disperse in fragmented forests. An increasing number of studies have demonstrated that plant assemblages in fragmented tropical landscapes can experience a process of floristic homogenization [40], [46], [47]. This process has been associated with ecological filters related to intensive land-use changes, and to the alteration of seed dispersal, seedling recruitment, and survival in fragmented landscapes (reviewed by Tabarelli et al. [48]). Our results thus suggest that changes in feeding strategies of spider monkeys in fragments can lead to the homogenization of the seed rain, which in turn could result in more homogeneous seedling carpets. A similar phenomenon may also occur with other key dispersers in fragments, intensifying the pattern we document with spider monkeys, but this remains to be tested. As spider monkeys are one of the most important dispersers of large-seeded species in these regions [19], [30], and fragmented forests continue to become more common in Neotropical landscapes, conservation and management efforts should concentrate on maintaining landscape connectivity. This action likely will help ameliorate the effects of homogenization of the seed rain and ultimately will help in assuring the maintenance of tropical ecosystems.

## Supporting Information

Figure S1 **Tree species diversity in continuous and fragmented forest sites in the Lacandona region, Mexico.** In panel (a) we indicate values for all trees with DBH >10 cm, whereas in panel (b) we show values for the top food tree species. Means (± SE) per site are indicated. In all cases, differences were not significant (*P*>0.05). In all cases, we evaluated true diversities of order 0 (species richness), 1 (exponential of Shannon's entropy), and 2 (inverse Simpson concentration).
(TIF)

Table S1 **Availability of top food tree species in continuous forest sites and fragmented forests in the Lacandona region, Mexico.** The total number of trees and total basal area (m^2^, in parentheses) is indicated for each tree species.
(DOC)

Table S2 **Seed species deposited by spider monkeys during a 13-mo period in 60 latrines located in two continuous forest sites and three forest fragments in the Lacandona region, Mexico.** The total number of seeds (and percentages, in parentheses) is indicated for each forest condition and for the entire landscape (i.e., considering both forest conditions).
(DOC)

Appendix S1 **Differences among sites and between forest conditions in vegetation composition and structure.**
(DOC)
