# Supplementary figures and images for: Contagious Deposition of Seeds in Spider Monkeys' Sleeping Trees Limits Effective Seed Dispersal in Fragmented Landscapes
Source: PLoS One. 2014 Feb 27;9(2):e89346. doi: 10.1371/journal.pone.0089346 (PMC3937327; doi:10.1371/journal.pone.0089346)

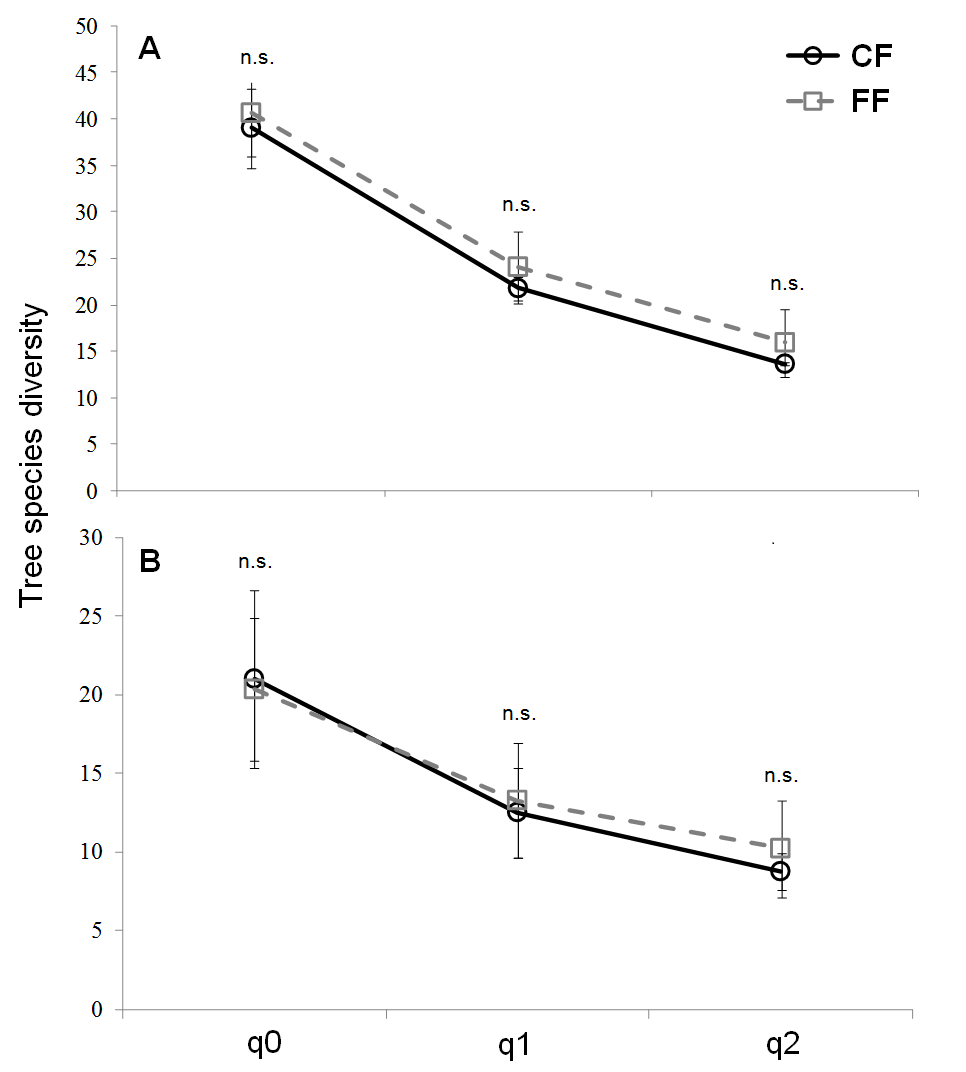

Supplement: Figure S1 — Tree species diversity in continuous and fragmented forest sites in the Lacandona region, Mexico. In panel (a) we indicate values for all trees with DBH >10 cm, whereas in panel (b) we show values for the top food tree species. Means (± SE) per site are indicated. In all cases, differences were not significant (P>0.05). In all cases, we evaluated true diversities of order 0 (species richness), 1 (exponential of Shannon's entropy), and 2 (inverse Simpson concentration). (TIF) [file pone.0089346.s001.tif]
