# Supplementary material for: Contagious Deposition of Seeds in Spider Monkeys' Sleeping Trees Limits Effective Seed Dispersal in Fragmented Landscapes
Source: PLoS One. 2014 Feb 27;9(2):e89346. doi: 10.1371/journal.pone.0089346 (PMC3937327; doi:10.1371/journal.pone.0089346)
Supplement: Table S1 — Availability of top food tree species in continuous forest sites and fragmented forests in the Lacandona region, Mexico. The total number of trees and total basal area (m2, in parentheses) is indicated for each tree species. (DOC) [file pone.0089346.s002.doc]

**Table S1**. Availability of top food tree species in continuous forest sites and fragmented forests in the Lacandona region, Mexico. The total number of trees and total basal area (m^2^, in parentheses) is indicated for each tree species.

| **Species** | **Family** | **CF1** | **CF2** | **FF1** | **FF2** | **FF3** |
| --- | --- | --- | --- | --- | --- | --- |
| *Ampelocera hottlei* | Ulmaceae | 8 (1.08) | 13 (0.39) | 0 | 2 (0.11) | 1 (0.04) |
| *Attalea butyracea* | Arecaceae | 0 | 0 | 0 | 7 (0.74) | 2 (0.29) |
| *Brosimum alicastrum* | Moraceae | 2 (0.50) | 7 (0.98) | 8 (0.66) | 7 (1.59) | 5 (1.79) |
| *Brosimum costaricanum* | Moraceae | 0 | 2 (0.05) | 0 | 0 | 0 |
| *Brosimum lactescens* | Moraceae | 0 | 0 | 0 | 3 (0.09) | 0 |
| *Bursera simaruba* | Burseraceae | 1 (0.00) | 0 | 0 | 7 (0.41) | 0 |
| *Castilla elastica* | Moraceae | 4 (0.29) | 10 (1.92) | 0 | 5 (0.15) | 7 (0.24) |
| *Cecropia obtusifolia* | Urticaceae | 1 (0.05) | 4 (0.19) | 0 | 0 | 1 (0.01) |
| *Coccoloba* sp. | Polygonaceae | 0 | 1 (0.20) | 0 | 0 | 0 |
| *Cojoba arborea* | Fabaceae | 4 (1.41) | 3 (0.28) | 0 | 0 | 1 (0.02) |
| *Cupania* sp. | Sapindaceae | 0 | 1 (0.03) | 0 | 0 | 0 |
| *Dialium guianense* | Fabaceae | 9 (1.16) | 13 (1.65) | 17 (1.14) | 18 (0.63) | 2 (0.19) |
| *Faramea occidentalis* | Rubiaceae | 0 | 0 | 0 | 0 | 1 (0.02) |
| *Ficus aurea* | Moraceae | 0 | 0 | 0 | 0 | 1 (0.97) |
| *Ficus* sp*.* | Moraceae | 4 (3.35) | 1 (1.99) | 0 | 1 (0.72) | 0 |
| *Guarea glabra* | Meliaceae | 18 (0.79) | 37 (0.85) | 11 (0.16) | 9 (0.29) | 13 (0.49) |
| *Guarea* sp. | Meliaceae | 0 | 0 | 3 (0.03) | 0 | 1 (0.01) |
| *Guarea grandifolia* | Meliaceae | 2 (0.06) | 0 | 1 (0.01) | 0 | 0 |
| *Hirtella americana* | Chrysobalanaceae | 0 | 0 | 0 | 1 (0.01) | 0 |
| *Inga* sp. | Fabaceae | 1 (0.01) | 0 | 0 | 1 (0.15) | 4 (0.13) |
| *Inga pavoniana* | Fabaceae | 1 (0.02) | 1 (0.01) | 0 | 1 (0.01) | 1 (0.04) |
| *Inga punctata* | Fabaceae | 0 | 1 (0.06) | 1 (0.03) | 0 | 0 |
| *Licania* sp. | Chrysobalanaceae | 0 | 2 (0.02) | 0 | 0 | 0 |
| *Licania platypus* | Chrysobalanaceae | 6 (0.92) | 4 (2.20) | 5 (1.18) | 4 (0.59) | 5 (2.56) |
| *Lonchocarpus* sp. | Fabaceae | 0 | 1 (0.06) | 1 (0.01) | 2 (0.13) | 0 |
| *Lonchocarpus cruentus* | Fabaceae | 0 | 0 | 0 | 2 (0.13) | 0 |
| *Mortoniodendron guatemalense* | Malvaceae | 2 (0.69) | 0 | 0 | 0 | 0 |
| *Nectandra ambigens* | Lauraceae | 0 | 3 (0.07) | 1 (0.13) | 0 | 4 (0.15) |
| *Nectandra* sp. | Lauraceae | 0 | 2 (0.03) | 0 | 1 (0.01) | 0 |
| *Poulsenia armata* | Moraceae | 0 | 0 | 0 | 0 | 5 (0.19) |
| *Pouteria* sp.1 | Sapotaceae | 7 (0.12) | 3 (0.06) | 0 | 9 (0.19) | 0 |
| *Pouteria campechiana* | Sapotaceae | 0 | 0 | 3 (0.09) | 1 (0.01) | 5 (0.22) |
| *Protium copal* | Burseraceae | 2 (0.04) | 6 (0.11) | 1 (0.01) | 0 | 0 |
| *Pterocarpus rohrii* | Fabaceae | 0 | 0 | 3 (0.80) | 1 (0.14) | 0 |
| *Quararibea funebris* | Malvaceae | 23 (0.81) | 15 (0.43) | 0 | 0 | 1 (0.05) |
| *Sabal mexicana* | Arecaceae | 0 | 0 | 19 (0.63) | 18 (0.64) | 0 |
| *Spondias mombin* | Anacardiaceae | 24 (4.66) | 0 | 1 (0.08) | 9 (0.47) | 1 (0.02) |
| *Spondias radlkoferi* | Anacardiaceae | 0 | 9 (1.75) | 2 (0.23) | 11 (1.38) | 6 (1.18) |
| *Tabebuia chrysantha* | Bignoniaceae | 0 | 0 | 1 (0.02) | 0 | 0 |
| *Tabebuia guayacan* | Bignoniaceae | 0 | 0 | 0 | 1 (0.07) | 0 |
| *Magnolia mexicana* | Magnoliaceae | 0 | 5 (0.49) | 0 | 0 | 0 |
| *Terminalia amazonia* | Combretaceae | 0 | 0 | 0 | 1 (0.13) | 0 |
| *Trichospermum mexicanum* | Malvaceae | 0 | 0 | 0 | 4 (0.21) | 0 |
| *Trophis mexicana* | Moraceae | 0 | 1 (0.05) | 0 | 0 | 0 |
| *Virola guatemalensis* | Myristicaceae | 0 | 4 (0.35) | 0 | 0 | 0 |
| Total | | 117 (16.0) | 149 (14.2) | 78 (5.2) | 126 (8.9) | 67 (7.6) |
