# Supplementary material for: Contagious Deposition of Seeds in Spider Monkeys' Sleeping Trees Limits Effective Seed Dispersal in Fragmented Landscapes
Source: PLoS One. 2014 Feb 27;9(2):e89346. doi: 10.1371/journal.pone.0089346 (PMC3937327; doi:10.1371/journal.pone.0089346)
Supplement: Table S2 — Seed species deposited by spider monkeys during a 13-mo period in 60 latrines located in two continuous forest sites and three forest fragments in the Lacandona region, Mexico. The total number of seeds (and percentages, in parentheses) is indicated for each forest condition and for the entire landscape (i.e., considering both forest conditions). (DOC) [file pone.0089346.s003.doc]

**Table S1**. **Seed species deposited by spider monkeys during a 13-mo period in 60 latrines located in two continuous forest sites and three forest fragments in the Lacandona region, Mexico**. The total number of seeds (and percentages, in parentheses) is indicated for each forest condition and for the entire landscape (i.e., considering both forest conditions).

| **Species** | **Family** | **Number (and percentage) of seeds** | | |
| --- | --- | --- | --- | --- |
|  |  | **Continuos**  **forest** | **Forest**  **fragments** | **Total** |
| *Sabal mexicana* | Arecaceae | 7 (0.0%) | 6,011 (18.1%) | 6,018 (13.1%) |
| *Dialium guianense* | Fabaceae | 486 (3.8%) | 5,308 (16.0%) | 5,794 (12.6%) |
| *Castilla elastica* | Moraceae | 1,479 (11.5%) | 2,771(8.3%) | 4,250 (9.2%) |
| *Spondias radlkoferi* | Anacardiaceae | 861 (6.7%) | 2,019 (6%) | 2,880 (6.2%) |
| *Trophis mexicana* | Moraceae | 835 (6.5%) | 1,559 (4.7%) | 2,394 (5.2%) |
| *Rourea glabra* | Connaraceae | 211 (1.6) | 2,150 (6.4%) | 2,361 (5.1%) |
| *Paullinia costata* | Sapindaceae | 1,328 (10.3%) | 822 (2.4%) | 2,150 (4.6%) |
| *Bactris mexicana* | Arecaceae | 2 (0.01%) | 1,665 (5%) | 1,667 (3.6%) |
| *Trichostigma octandrium* | Phytolaccaceae | 1,393 (10.8%) | 267 (0.8%) | 1,660 (3.6%) |
| *Ampelocera hottlei* | Ulmaceae | 1,316 (10.2%) | 320 (0.9%) | 1,636 (3.5%) |
| *Mendoncia retusa* | Acanthaceae | 1,313 (10.2%) | 293 (0.8%) | 1,606 (3.4% |
| *Nectandra ambigens* | Lauraceae | 123 (0.9%) | 1294 (3.9%) | 1,417 (3.0%) |
| *Spondias mombin* | Anacardiaceae | 35 (0.2%) | 1162 (3.5%) | 1,197 (2.6%) |
| *Acacia cornígera* | Fabaceae | 33(0.2%) | 856 (2.5%) | 889 (1.9%) |
| *Bactris americana* | Arecaceae | 1 (0%) | 875 (2.6%) | 876 (1.9%) |
| *Celtis iguanea* | Cannabaceae | 188 (1.4%) | 670 (2%) | 858 (1.8%) |
| Arecaceae (1) | Arecaceae | 42 (0.3%) | 727 (2.1%) | 769 (1.6%) |
| *Virola guatemalensis* | Myristicaceae | 588 (4.5%) | 44 (0.1%) | 632 (1.3%) |
| Morphoespecie (3) | - | 340 (2.6%) | 279 (0.8%) | 619 (1.3%) |
| *Desmoncus ortacanthus* | Arecaceae | 49 (0.3%) | 496 (1.4%) | 545 (1.1%) |
| *Guarea glabra* | Meliaceae | 323 (2.5%) | 192 (0.5%) | 515 (1.1%) |
| *Attalea butyraceae* | Arecaceae | 25 (0.1%) | 466 (1.4%) | 491 (1.0%) |
| *Theobroma cacao* | Malvaceae | 0 | 470 (1.4%) | 470 (1.0%) |
| *Brosimum lactescens* | Moraceae | 162 (1.2%) | 254 (0.7%) | 416 (0.9%) |
| Morphoespecie (2) | - | 388 (3.0%) | 0 | 388 (0.8%) |
| *Serjania* sp. | Sapindaceae | 2 (0.0%) | 255 (1.0%) | 357 (0.6%) |
| *Inga* sp. | Fabaceae | 93 (0.7%) | 259 (0.7%) | 352 (0.6%) |
| *Faramea occidentalis* | Rubiaceae | 18(0.1%) | 282 (0.8%) | 300 (0.3%) |
| Morphoespecie (1) | - | 129(1%) | 129 (0.3%) | 258 (0.5%) |
| *Smilax* sp. | Smilacaceae | 0 | 252 (0.7%) | 252 (0.5%) |
| Morphoespecie (5) | - | 166 (1.2%) | 0 | 166 (0.3%) |
| *Abuta panamensis* | Menispermaceae | 142(1.1%) | 20 (0.0%) | 162 (0.3%) |
| Vitaceae | Vitaceae | 0 | 137 (0.4%) | 137 (0.2%) |
| *Syngonium* sp*.* | Araceae | 59(0.4%) | 72 (0.21%) | 131 (0.2%) |
| *Rollinia* sp. | Annonaceae | 123(0.9%) | 0 | 123 (0.2%) |
| *Strichnos tabascana* | Loganiaceae | 0 | 114 (0.3%) | 114 (0.2%) |
| *Posoqueria latifolia* | Rubiaceae | 27(0.2%) | 81 (0.2%) | 108 (0.2%) |
| Lauraceae | Lauraceae | 0 | 106(0.3%) | 106 (0.2%) |
| Morphoespecie (4) | - | 0 | 105(0.3%) | 105 (0.2%) |
| *Cissus microcarpa* | Vitaceae | 77(0.6%) | 25(0.07%) | 101 (0.2%) |
| *Chionantus dominguensis* | Oleaceae | 78(0.6%) | 21(0.06%) | 99 (0.2%) |
| *Garcinia intermedia* | Clusiaceae | 18(0.1%) | 71(0.2%) | 89 (0.1%) |
| *Hirtella americana* | Chrysobalanaceae | 67(0.5%) | 0 | 67 (0.1%) |
| *Inga punctata* | Fabaceae | 31(0.2%) | 16(0.04%) | 47 (0.1%) |
| *Guarea grandifolia* | Meliaceae | 41(0.3%) | 1(0.0%) | 42 (0.9%) |
| *Bursera simaruba* | Burseraceae | 15(0.1%) | 22(0.06%) | 37 (0.8%) |
| *Chamaedorea tepejilote* | Arecaceae | 0 | 34(0.1%) | 34 (0.7%) |
| *Cupania* sp. | Sapindaceae | 34(0.2%) | 0(0%) | 34 (0.7%) |
| *Cissus* sp. | Vitaceae | 26(0.2%) | 0(0%) | 26 (0.05%) |
| Arecaceae (2) | Arecaceae | 23(0.1%) | 0(0%) | 23 (0.05%) |
| Araceae | Araceae | 8(0.06%) | 13(0.03%) | 21(0.04%) |
| *Pseudolmedia glabrata* | Moraceae | 21(0.1%) | 0 | 21 (0.04%) |
| Arecaceae (3) | Arecaceae | 16(0.1%) | 0 | 16 (0.03%) |
| *Trophis racemosa* | Moraceae | 0 | 13 (0.03%) | 13 (0.03%) |
| *Mollinedia* sp. | Monimiaceae | 12(0.09%) | 0 | 12 (0.02%) |
| *Guazuma ulmifolia* | Malvaceae | 0 | 10 (0.03%) | 10 (0.02%) |
| *Clarisia racemosa* | Moraceae | 8(0.06%) | 0 | 8 (0.01%) |
| *Monstera* sp. | Araceae | 0 | 8(0.02%) | 8 (0.01%) |
| *Psichotrya chiapensis* | Rubiaceae | 0 | 8(0.02%) | 8 (0.01%) |
| *Tetracera* sp. | Dilleniaceae | 0 | 7(0.02%) | 7 (0.01%) |
| *Myrtaceae* | Myrtaceae | 6(0.04%) | 0 | 6 (0.01%) |
| *Anturio* sp. | Araceae | 5(0.03%) | 0 | 5 (0.01%) |
| *Calatola* sp. | Icacinaceae | 5(0.03%) | 0 | 5 (0.01%) |
| *Licaria hipoleuca* | Lauraceae | 5(0.03%) | 0 | 5 (0.01%) |
| *Passiflora helleri* | Passifloraceae | 0 | 2(0.0%) | 2 (0%) |
| *Magnolia mexicana* | Magnoliaceae | 2(0.01%) | 0 | 2 (%) |
| *Cymbopetalum pendiflorum* | Annonaceae | 0 | 1(0.0%) | 1 (%) |
| *Pouteria* sp. | Sapotaceae | 0 | 1(0.0%) | 1 (%) |
