# Supplementary material for: Contagious Deposition of Seeds in Spider Monkeys' Sleeping Trees Limits Effective Seed Dispersal in Fragmented Landscapes
Source: PLoS One. 2014 Feb 27;9(2):e89346. doi: 10.1371/journal.pone.0089346 (PMC3937327; doi:10.1371/journal.pone.0089346)
Supplement: Appendix S1 — Differences among sites and between forest conditions in vegetation composition and structure. (DOC) [file pone.0089346.s004.doc]

**Appendix S1. Differences among sites and between forest conditions in vegetation composition and structure**

To assess if differences in seed species diversity between forest conditions could be related to inter-forest variations in vegetation structure, we sampled vegetation within each site following the Gentry [1] protocol. We randomly located twenty 50 x 2-m transects (2,000 m^2^) and identified and measured the diameter at breast height (dbh) of all trees with dbh ≥ 10 cm. We chose this method because it has been used to describe the habitat of other Mexican primates (e.g., *Alouatta palliata* [2,3,4], *Ateles geoffroyi* [5]), and hence, it is possible to compare our data with other Mexican sites.

For each site (i.e., pooling the transect data for each site), we quantified tree species diversity using true diversity measures of order 0, 1 and 2 (see the text for further details on these metrics). Additionally, using data from a recent review of spider monkey’s diet in Mesoamerica [6], we identified all plant species that together constituted > 80% of total feeding time (TFT) in this review paper and that were present in our study transects. We considered these species as potential top food species contributing to resource abundance, and we then calculated true diversities of order 0, 1 and 2 for these top food tree species. Additionally, we calculated total stem density and total basal area of top food species within each site.

The differences between forest conditions in tree species diversity, and in density and basal area of top food species were tested with generalized linear models. As suggested for count dependent variables (i.e., diversity of order 0 and density of top food trees), we used a Poisson error and a log link function. For the basal area of top foods and for the diversities of order 1 and 2 we used normal error and identity link function [7].

Both forest conditions showed a similar number of tree species (Figure S1a). This was evident when considering rare (i.e., ^0^D, species richness) (*χ^2^* = 0.08, df = 1, *P* = 0.77), common (i.e., ^1^D, exponential of Shannon’s entropy) (*χ^2^* = 1.02, df = 1, *P* = 0.31), and dominant (^2^D, inverse Simpson concentration) tree species (*χ^2^* = 1.67, df = 1, *P* = 0.27). When assessing the top food tree species, tree species diversity was also similar in both forest conditions when considering ^0^D (χ^2^ = 0.02, df = 1, *P* = 0.087), ^1^D (χ^2^ = 0.09, df = 1, *P* = 0.75) and ^2^D (χ^2^ = 0.62, df = 1, *P* = 0.43) (Figure S1b). However, tree density of top food species was higher in continuous forest (mean ± SE, 133 ± 22 stems/2,000 m^2^) than in forest fragments (90 ± 31 stems/2,000 m^2^; *χ^2^* = 104.4, df = 1, *P* < 0.001). The basal area of top food species was also higher in continuous (0.73 ± 0.22 m^2^) than in fragmented forest sites (0.35 ± 0.02 m^2^) (*χ^2^* =15.9, df = 1, *P* < 0.001).

**References**

1. Gentry AH (1982) Patterns of Neotropical plant species diversity. Evol Biol 15:1–84.

2. Arroyo-Rodríguez V, Mandujano S (2006) Forest fragmentation modifies habitat quality for *Alouatta palliata*. Int J Primatol 27:1079–1096.

3. Arroyo-Rodríguez V, Mandujano S, Benítez-Malvido J, Cuende Fanton C (2007) The influence of large tree density on howler monkey (*Alouatta palliata mexicana*) presence in very small rain forest fragments. Biotropica 39:760–766.

4. Dunn JC, Cristóbal-Azkarate J, Vea J (2009) Differences in diet and activity pattern between two groups of *Alouatta palliata* associated with the availability of big trees and fruit of top food taxa. Am J Primatol 71:654–662.

5. Chaves OM, Stoner KE, Arroyo-Rodríguez V (2012) Differences in diet between spider monkey groups living in forest fragments and continuous forest in Lacandona, Mexico. Biotropica 44:105–113.

6. González-Zamora A, Arroyo-Rodríguez V, Chaves OM, Sánchez S, Stoner KE, Riba-Hernández P (2009) Diet of spider monkeys (*Ateles geoffroyi*) in Mesoamerica: Current knowledge and future directions. Am J Primatol 71:8–20.

7. Crawley M (2002) Statistical computing: An introduction to data analysis using S-Plus. Chichester: John Wiley and Sons, 772 p.
